# Innexin 3, a New Gene Required for Dorsal Closure in *Drosophila* Embryo

**DOI:** 10.1371/journal.pone.0069212

**Published:** 2013-07-24

**Authors:** Fabrizio Giuliani, Giuliano Giuliani, Reinhard Bauer, Catherine Rabouille

**Affiliations:** 1 Hubrecht Institute for Developmental Biology and Stem Cell Research, Utrecht, The Netherlands; 2 UMC Utrecht, Utrecht, The Netherlands; 3 LIMES-Institute, Program Unit Development, Genetics and Molecular Physiology, Laboratory for Molecular Developmental Biology, University of Bonn, Bonn, Germany; 4 Department of Cell Biology, UMC Utrecht, Utrecht, The Netherlands; University of North Carolina at Chapel Hill, United States of America

## Abstract

**Background:**

Dorsal closure is a morphogenetic event that occurs during mid-embryogenesis in many insects including *Drosophila,* during which the ectoderm migrates on the extraembryonic amnioserosa to seal the embryo dorsally. The contribution of the ectoderm in this event has been known for a long time. However, amnioserosa tension and contractibility have recently been shown also to be instrumental to the closure. A critical pre-requisite for dorsal closure is integrity of these tissues that in part is mediated by cell-cell junctions and cell adhesion. In this regard, mutations impairing junction formation and/or adhesion lead to dorsal closure. However, no role for the gap junction proteins Innexins has so far been described.

**Results and Discussion:**

Here, we show that Innexin 1, 2 and 3, are present in the ectoderm but also in the amnioserosa in plaques consistent with gap junctions. However, only the loss of Inx3 leads to dorsal closure defects that are completely rescued by overexpression of *inx3::GFP* in the whole embryo. Loss of Inx3 leads to the destabilisation of Inx1, Inx2 and DE-cadherin at the plasma membrane, suggesting that these four proteins form a complex. Accordingly, in addition to the known interaction of Inx2 with DE-cadherin, we show that Inx3 can bind to DE-cadherin. Furthermore, Inx3-GFP overexpression recruits DE-cadherin from its wildtype plasma membrane domain to typical Innexin plaques, strengthening the notion that they form a complex. Finally, we show that Inx3 stability is directly dependent on tissue tension. Taken together, we propose that Inx3 is a critical factor for dorsal closure and that it mediates the stability of Inx1, 2 and DE-cadherin by forming a complex.

## Introduction

*Drosophila* embryogenesis consists of a series of very dynamic and highly regulated morphogenetic events through which a series of orchestrated cellular movements and rearrangements occur, including the morphogenetic process termed dorsal closure that seals the embryo. At the end of germband retraction (embryonic stage 12) a large hole is left on the dorsal surface of the ectoderm that is covered by the amnioserosa, a sheet of flat and large epithelial extraembryonic cells. At stage 13, the two opposing ectodermal epithelial cell sheets start to migrate over the amnioserosa. The ectodermal leading edge cells begin to elongate along their dorsoventral axis. This is followed by filopodia extension and lateral elongation (spreading) resulting in the epithelial cells from opposite sides of the embryo meeting at the dorsal midline to form junctions, thus allowing the hole to close to form a seamless dorsal midline at stage 15 (12 hours after egg lay, AEL) [1].

Although, for a long time, the ectoderm was thought to be the sole actor in dorsal closure, recent work has shown that the contribution of the amnioserosa is also essential [2], [3]. First, before and during dorsal closure, the amnioserosa has been shown to undergo a series of rapid contractions, so called “pulse” [4]. Second, these pulses have been shown to be necessary for dorsal closure and interference in this pulsing event, for instance by laser ablation, delays significantly the closure [4], [5]. The emerging model is that the spatial temporal co-ordination of forces emanating from both the ectoderm and the amnioserosa are essential for proper dorsal closure. The movement of the ectoderm over the amnioserosa is due to the contractile force of an actomyosin-rich supracellular “purse-string” actin cable located at the leading edge of the migrating ectodermal cells surrounding the hole and the critical pulling force generated by the cortical actomyosin networks in amnioserosa cells. Taken together, these observations show that dorsal closure depends on the tension in the amnioserosa.

In this regard, the junctions between the aminoserosa cells are clearly important in dorsal closure, as they maintain tissue integrity critical for tissue tension and allow the pulsatile activity of the amnioserosa. In fact, the overexpression of the transcription factor grainyhead in the amnioserosa that leads, among others, to the expression of genes encoding septate junction proteins also results in dorsal closure defects [6]. One interpretation of these defects is that additional junction proteins alter the adhesive properties of the amnioserosa that could impair the pulsatile activity of the amnioserosa.

A number of junctional proteins are known to function in this tissue. One is Crumbs, an apical transmembrane protein normally required for organizing apical-basal polarity. Adherens junctions are also present and are characterized by the presence of the transmembrane protein DE-cadherin [7], [8]. Septate junctions are absent in agreement with the lack of expression of septate junction proteins [9], [10], [11], [12]. At the basal side, the amnioserosa expresses integrins that mediate cell-extracellular matrix adhesion between the amnioserosa and the underlying yolk [13], [14], [15], [16], [17], [5]. Accordingly, mutations in *crumbs*, *shotgun* (encoding DE-cadherin) and *myspheroid* (encoding the βPS integrin subunit) result in dorsal closure defects [18], [7], [8], [19], [20], [5]. Whether gap junctions are present in this tissue and play a role in dorsal closure is not known.

Gap junctions consist of a tightly packed array of intercellular channels between adjacent cells that facilitate passage of small molecules, such as cAMP and inositol triphosphate, ions and signaling peptides from one cell to the next [21], [22], [23]. In vertebrates, these channels are made of hexameric rings, known as hemichannels, one on each opposing cell surface of two adjacent cells, that dock to form the complete intercellular gap junction channel [24]. These hemichannels are made of 6 homo or heterohexamers of connexins, a family of protein spanning the membrane 4 times with 2 extracellular loops and cytosolic N- and C-termini. Hemichannels can also comprise hexamers of pannexins that are structurally related to connexins but do not share significant sequence homology. Three pannexin-encoding genes are present in both the human and mouse genomes. For a long time, the 3 mammalian pannexins were considered to form gap junction-like structures. However, several reports have demonstrated that, unlike the connexin gap junctions intercellular channels, pannexin oligomers are channels embedded primarily in a single plasma membrane that, when open, allow a conduction pathway between the cytosol and extracellular space [25].

In invertebrates, the building blocks that compose gap junctions are termed Innexins (Inx) that bear significant amino-acid sequence similarity to the 3 mammalian pannexins and no homology to connexins [26], [27], [28]. However, innexins can form gap junction channels, a property they share with connexins. There are 8 *innexin* genes present in the *Drosophila* genome, out of which seven are differentially expressed in the central nervous system (CNS) [29], including *inx2* and *3*. *inx1/ogre* mutants exhibit defects in the optic ganglia size and signal transmission at electrical synapses of the giant fiber system [30], [31], [32] and *inx7* is implicated in axon guidance and embryonic nervous system development [33].

In addition to their expression in the CNS, innexins have been also shown to function during embryogenesis, where they form gap junctions at very early stages of development [34], [35]. Inx2 is the most studied member of the Innexin family in embryonic development, where it is required for cell polarity and epithelial tissue organization [36], [34], [35]. In addition, Inx2 is essential for the development of the proventriculus in the posterior foregut region of the *Drosophila* embryo [37]. In this context, Inx2 has been shown to form hemichannels with Inx3 in the epidermis of the developing embryo. They interact biochemically and Inx3 is no longer deposited at the plasma membrane in *inx2/kropf* mutant embryos. Furthermore *inx3* RNAi leads to *inx2*-like mutant phenotype, including mislocalization of Inx2 and DE-cadherin [35]. This suggests that heteromerization of Inx2 and 3 is crucial for epithelial organization and polarity of the embryonic epidermis [36], [34], [35].

However, to date, there is no description of gap junction involvement in dorsal closure. Here, we analyze the expression and the loss of function phenotype of 3 members of the innexin family during dorsal closure stages. We find that Inx1, 2 and 3 are expressed in the ectoderm and in the aminoserosa cells of the developing embryo during mid-embryogenesis. However, only loss of Inx3 zygotic function leads dorsal closure defects.

## Results and Discussion

### The gap junction proteins Inx1, Inx2 and Inx3 are expressed in the amnioserosa and ectoderm during dorsal closure stages

**Figure 1:**
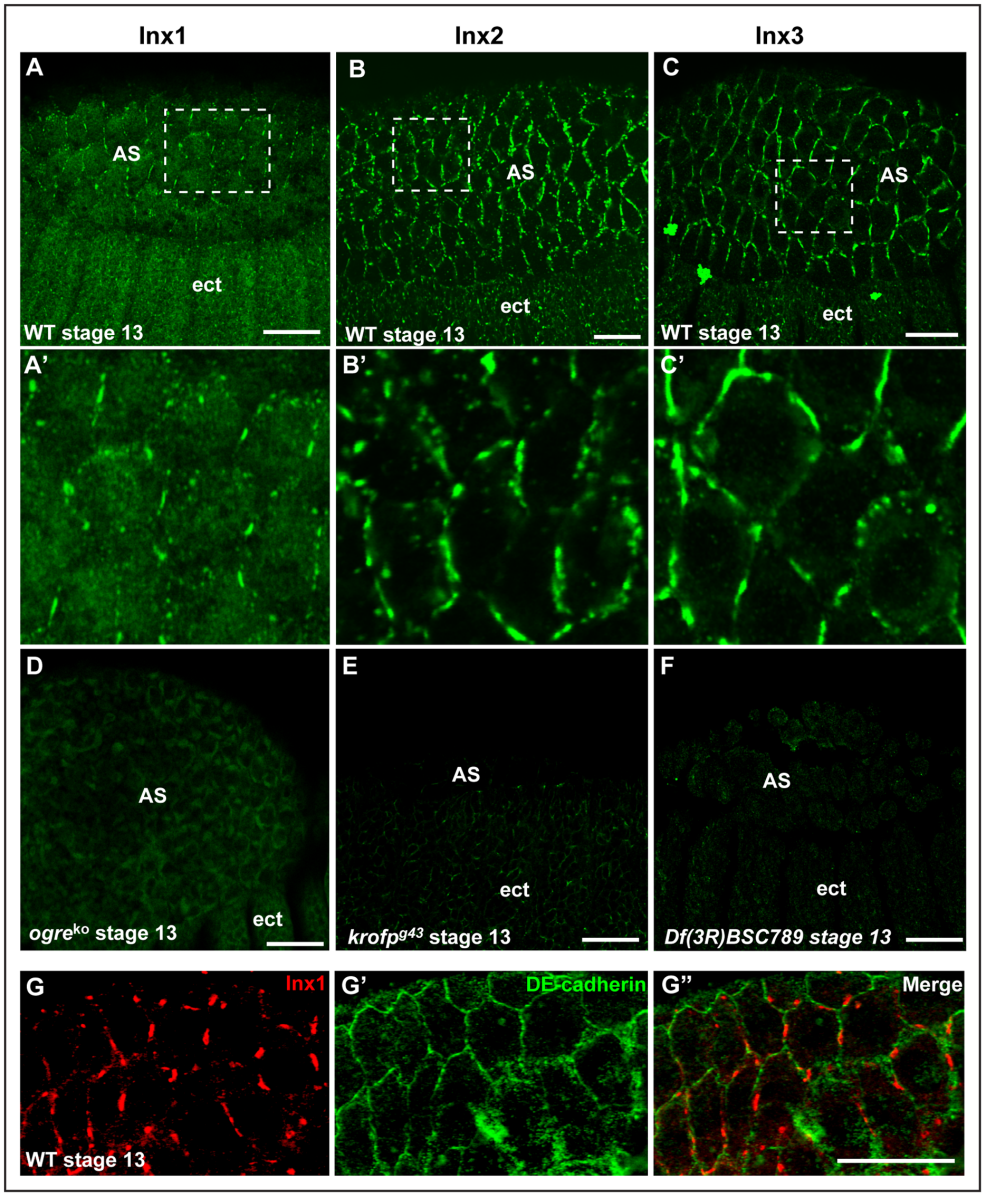
Innexin 1,2,3 localize to the plasma membrane of ectodermal and amnioserosa cells. A–C: Immunolocalisation of endogenous Inx1 (A), Inx2 (B) and Inx3 (C) in wildtype (WT) *Drosophila* embryos (stage 11–13). **A'–C'**: Details of Inx1-3 localisation in plaques at the plasma membrane of the amnioserosa cells (blowup from dashed areas in A–C) **D–F**: Immunolocalisation of Inx1 in *ogre* ^ko^ (D), Inx2 in *kropf* ^g43^ (E) and Inx3 in *Df(3R)BSC789* (F) homozygous mutants, showing the antibodies specificity. The lack of labeling is not due to loss of tissue (see Figure 2 for *ogre* ^ko^ and *kropf* ^g43^ and Figure 4 for *Df(3R)BSC789)*. **G–G”**: Immunolocalisation of endogenous Inx1 (G, red) and DE-cadherin (G', green) in amnioserosa cells of WT stage 13 embryos. Scale bars: 25 µm.

In order to address the role of gap junctions in dorsal closure, we first assessed the localisation of Inx1, 2, 3 and 7 in dorsal closure stages of *Drosophila* embryogenesis using specific antibodies raised against the different proteins (Figure 1D-F). In addition to their previously described expression in the ectoderm (see introduction), we found that Inx1 (Figure 1A and Fig S1A), Inx2 (Figure 1B and Fig S1B) and Inx3 (Figure 1C and Fig S1C), but not Inx7 (not shown), are also expressed from stages 11 to 15, not only in the ectoderm but also in the amnioserosa cell plasma membrane where they form a classical pattern of “plaques” characteristics of gap junctions (Figure 1A'–C'). Double staining with the adherens junctional marker DE-cadherin indicates that gap junctions reside in a subdomain of adherens junctions (Figure 1G–G'').

### Loss of Inx3 leads to dorsal closure defects

**Figure 2:**
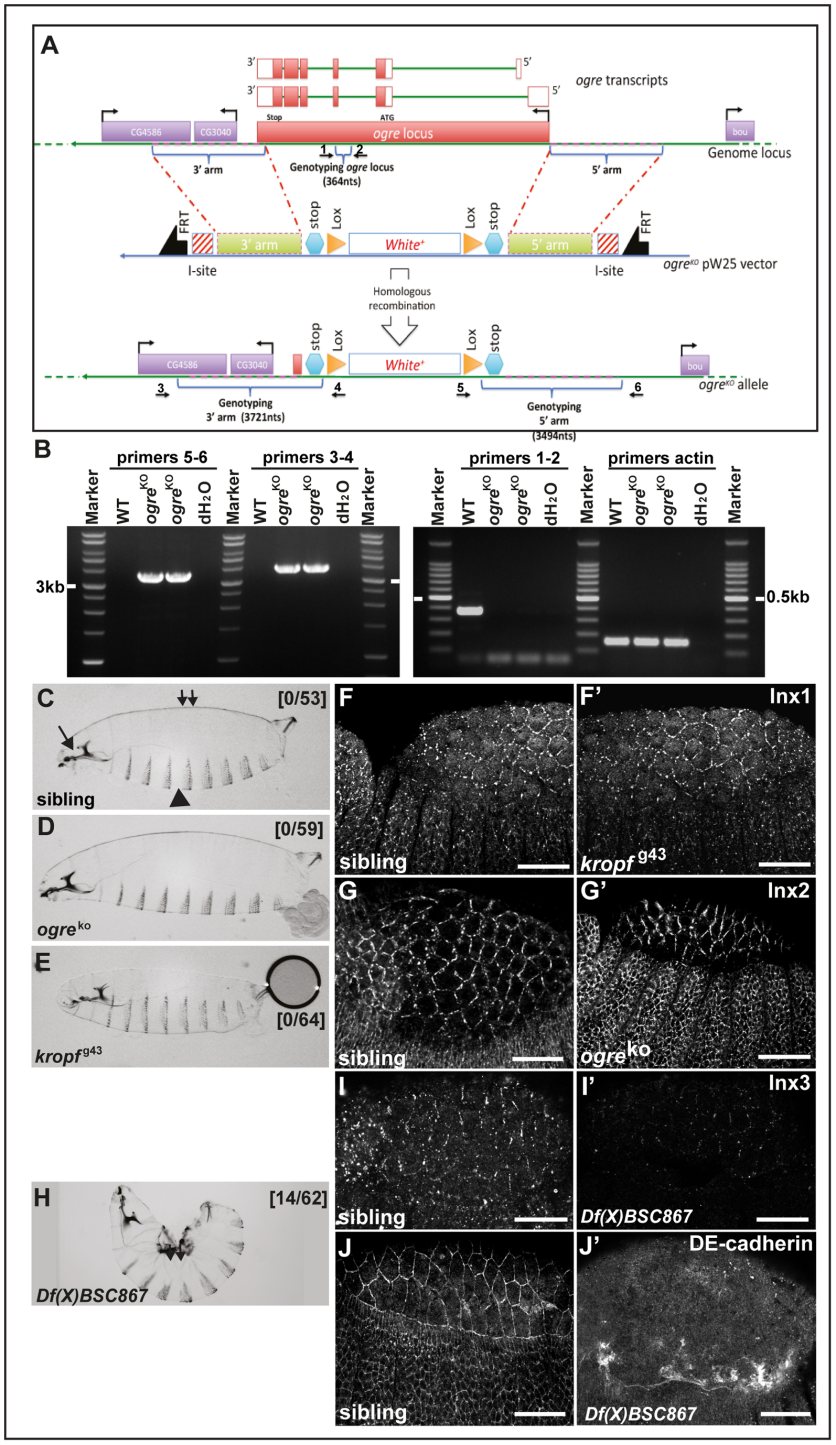
Zygotic loss of *inx1* and *2* do not lead to dorsal closure defects. A: Schematic representation of the genomic locus (top), transcripts (middle) and targeting vector of *ogre/inx1*. The centromere is to the right of this genomic fragment. The transcript is drawn to scale with the genomic diagram, with exons depicted as rectangles and coding regions filled with black. Introns are depicted as bent lines below the exons. The five coding exons of the *ogre* gene are numbered. Positions of primers used for PCR in (B) are indicated with arrows and numbers (1–6). **B**: PCR products of genomic DNA from wildtype (OreR) and *ogre^ko^* homozygous flies using 3 sets of primers. Combination 3–4 yields an expected band at 3.9 kb in WT but not in *ogre^ko^*. Combination 5–6 yields an expected band of 3.7 kb and combination 1–2 yields a band of 364 bp. A product of the predicted size is generated only with genomic DNA from mutant flies, because primer 1 sequence is located outside the 5′ arm of the targeting vector, and primer 2 sequences is within the *w+* gene. **C–E**: Brightfield micrographs of dorso-lateral cuticle preparations from wildtype (C) showing normal cuticular features including the dorsal side (small double arrow), ventral denticle belts (arrowhead) and head skeleton (arrow), *ogre^ko^/inx1 (D)*, *kropf^g43^/inx2* (E) first instar larvae. Note the absence of dorsal closure defects, Numbers in brackets design the ratio of the numbers of larvae with dorsal closure defects over the total of larvae examined. Sibling indicates siblings in each line. Anterior left; dorsal up. **F–G'**: Immunolocalisation of endogenous Inx2 in *ogre^ko^* and of Inx1 in *kropf* ^g43^ in stage 13 homozygous mutant embryos. **H:** Brightfield micrographs of dorso-lateral cuticle preparations from homozygous *Df(X)BSC867* uncovering the *inx1-2–7* locus that exhibits a severe kink and holes (small double arrow). **I–J':** Immunolocalisation of endogenous Inx3 (I, I') and DE-cadherin (J, J') in hererozygous (I, J) and homozygous *Df(X)BSC867* embryos (I', J'). Scale bars: 25 µm.

To test whether the localisation of Inx1, 2 and 3 is functional in dorsal closure, we analysed the loss of function phenotypes of these 3 proteins. In the absence of an available *ogre/inx1* mutant, we generated an RNA null allele of *ogre* by homologous recombination, *ogre* ^ko^, in which the ORF was completely replaced by the mini-white gene (Figure 2A, B) [38]. The *ogre* ^ko^ is a viable allele and homozygous mutant larvae do not display any dorsal closure defects (Figure 2D) when compared to siblings (Figure 2C).

For *inx2*, we used the available EP line *kropf* ^g43^ that disrupts the *inx2* locus (Bloomington). *inx2* locus lies within the first intron of *inx7* and the expression of the latter is unaffected in *kropf* ^g43^ allele (Flybase). As reported, *kropf^g43^* mutant hemizygotes die at first instar larva stage but they develop normal cuticles [34] (Figure 2E). In agreement, loss of Inx1 does not lead to defects in Inx2 expression and localisation and *vice versa* (Figure 2F–G'), indicating that Inx1 and 2 are not involved in dorsal closure or that they are redundant. To test this, we could generate *ogre kropf* double mutant embryos. However, the two loci lie physically too close to each other to efficiently obtain a double mutant combination by recombination. To circumvent this issue, we made use of the *Df(X)BSC867* (6E4;6F1) that uncovers the *ogre, kropf and inx7* loci. *Df(X)BSC867* hemizygous larvae exhibit severe dorsal closure defects (Figure 2H), suggesting that the combined function of Inx1 and 2 may be critical for this morphogenetic event. However, this dorsal closure phenotype cannot be attributed to the sole effect of the loss of expression of Inx1 and 2, as *Df(X)BSC867* deficiency also uncovers many other genes, including *boudin* that encodes a septate junction component [39], (Flybase).

**Figure 3:**
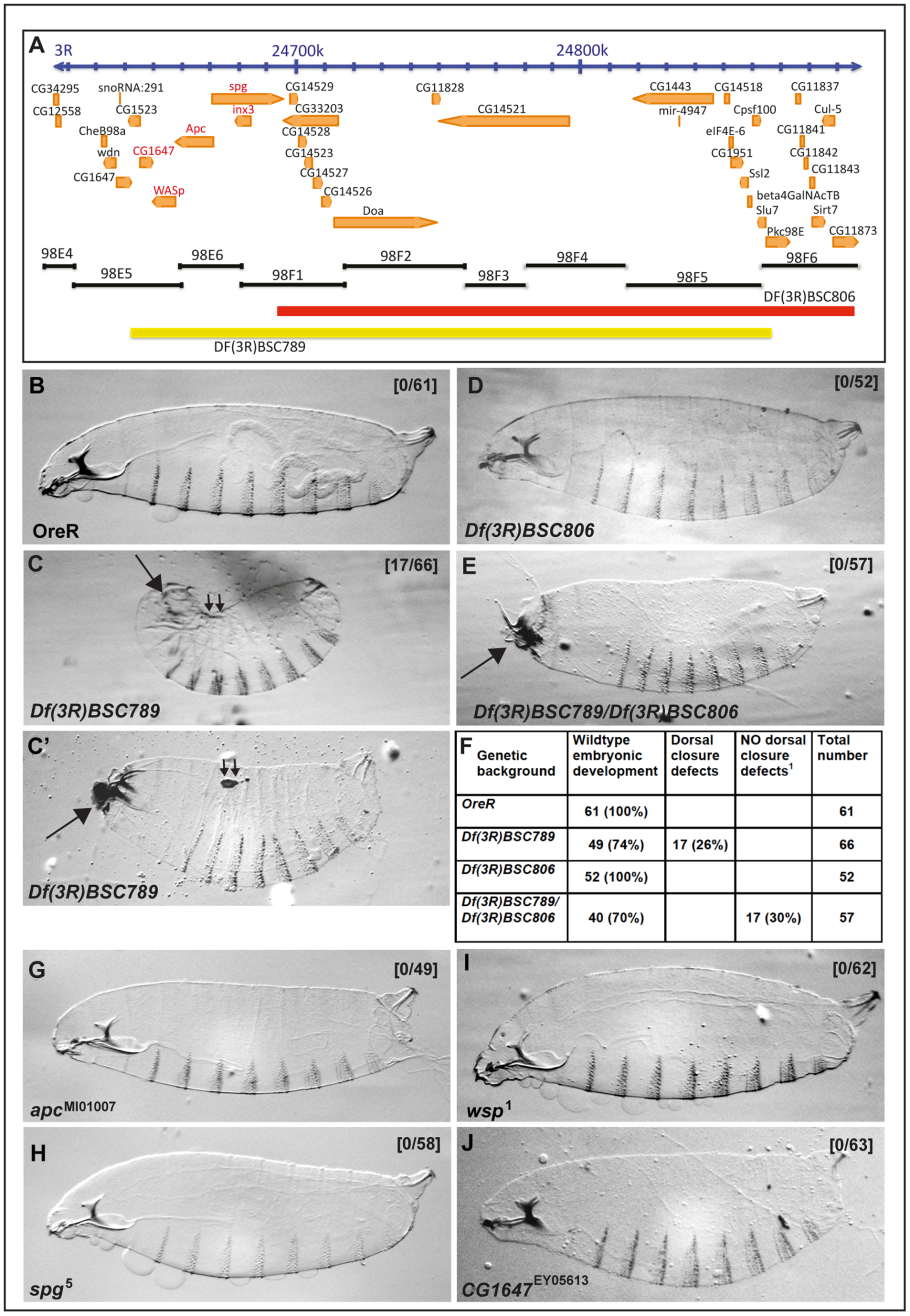
Loss of *inx3* gives strong dorsal closure defects. A: Schematic illustration of the genomic region uncovered by *Df(3R)BSC789* and *Df(3R)BSC806*. **B–C'**: Brightfield micrographs of dorso-lateral cuticle preparation of *OreR* (B) and *Df(3R)BSC789* (C–C') first instar larvae. Note small holes on the dorsal side of the embryo in (small double arrows in C') and the kink (small double arrows in C). **D–E:** Brightfield micrographs of dorso-lateral cuticle preparation of the larval progeny of *Df(3R)BSC806* (D), of the cross of *Df(3R)BSC789* with *Df(3R)BSC806* (E). **F**: Quantification of the dorsal closure defects in larvae of the same background as above in B to E. **G–J:** Brightfield micrographs of dorso-lateral cuticle preparation of the larval progeny of *apc* (*apc* ^1^, G), *sponge* (*spg* ^5^, H), *wasp* (*wsp* ^1^, I), and *CG1647* (J). Note the absence of dorsal closure defects in 100% of the larvae. Numbers in brackets design the ration of the numbers of larvae with dorsal closure defects over the total of larvae examined.

In the absence of an *inx3* mutant allele or any available P-element insertion in the proximity of the inx3 gene, we first used the *Df(3R)BSC789* deficiency (98E5-98F6) uncovering the *inx3* locus (Figure 3A). *Df(3R)BSC789* is embryonic lethal and homozygous embryos produce cuticles with strong head involution (arrow in Figure 3C and C') and dorsal closure defects, including kinked features (small double arrows in Figure 3C,) and holes (small double arrows in Figure 3C') in the dorsal epidermis (Figure 2F). On the other hand, the ventro-lateral epidermis and the denticle belts were unaffected (Figure 3B–C').

Considering the large number of genes uncovered by *Df(3R)BSC789*, to pin-point whether this dorsal closure phenotype is due to the loss of *inx3* gene, we use of a second deficiency, *Df(3R)BSC806* (98F1-98F10), that spans most of the genes also encompassed by *Df(3R)BSC789*, with the exception of a small genomic region around *inx3* (Figure 3A). Cuticle preparations of *Df(3R)BSC806* homozygous and *Df(3R)BSC789/Df(3R)BSC806* trans-heterozyous first instar larvae did not display any dorsal closure defects (Figure 3D–F), although the head development was still affected (Figure 3E). This indicates that the locus responsible for the dorsal closure defects is situated in a genomic region that contains five genes (marked in red in Figure 3A): *wasp (wsp)*, *apc1, inx3*, *sponge (spg)* and *CG1647*.

Previous reports have shown that mutations in *apc*, *spg* and *wsp* do not lead to dorsal closure defects [40], [41], [42], [43], and we further confirmed this by examining and quantifying cuticle preparations from these mutants (Figure 3G–J). On the other hand, *CG1647* is an uncharacterized locus that is predicted to encode a zinc finger containing protein. To assess whether the loss of function of this gene causes dorsal closure defects, we used the EY05613 line where the *CG1647* locus is disrupted by a P-element insertion in the exon 3 (Flybase). The EY05613 allele is viable and homozygous mutant larvae developed perfectly normal cuticles (Figure 3J).

**Figure 4:**
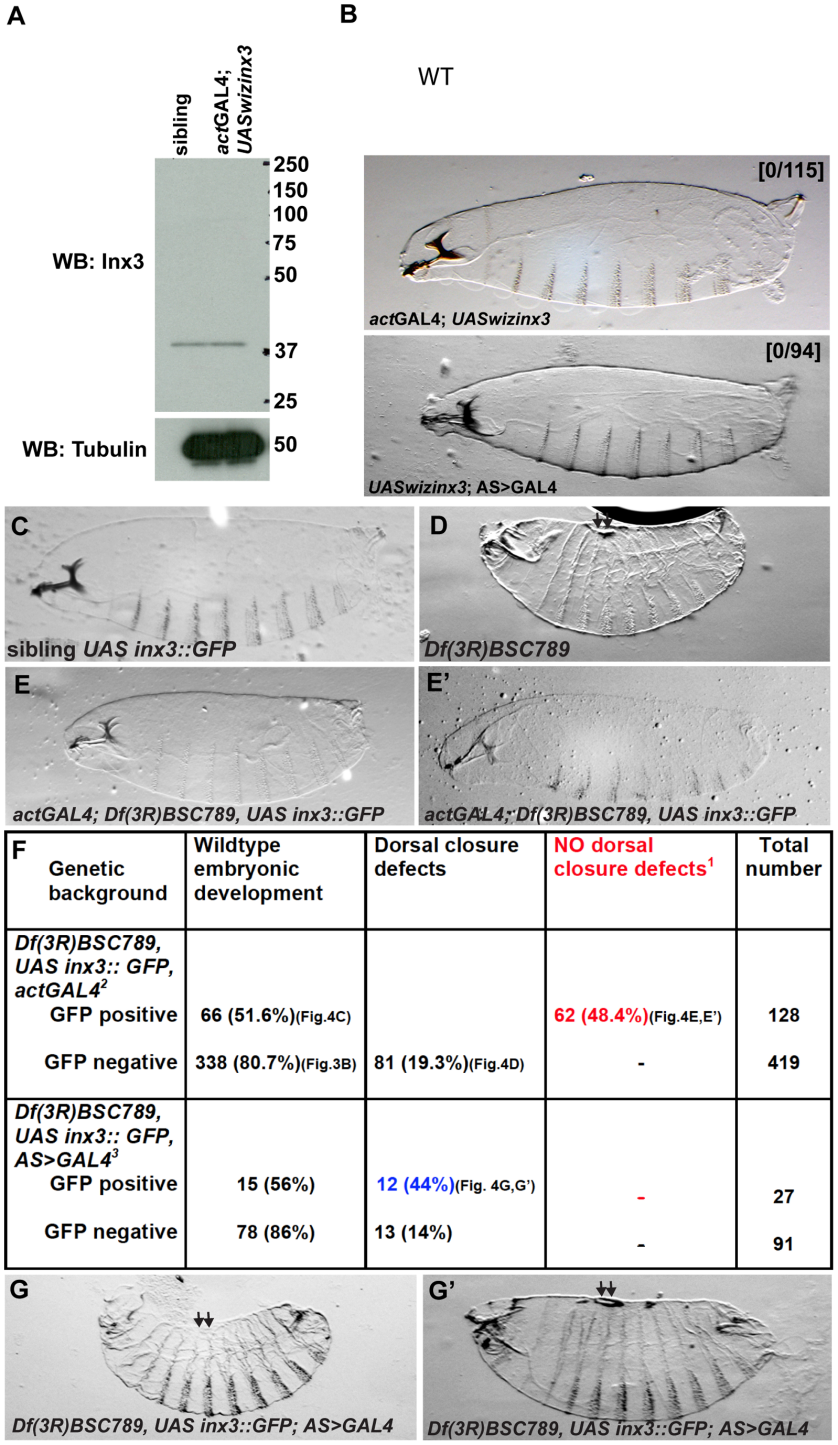
*inx3::GFP* overexpression rescues dorsal closure defects of *Df(3R)BSC789*. A: Western blot detection of Inx3 upon Inx3 depletion by RNAi using *UASwizinx3* and *actGAL4*. Note that the overexpression of *UASwizinx3* is not enough to deplete Inx3 when compared to siblings. **B, B'**: Brightfield micrographs of dorso-lateral cuticle preparation of *actGAL4*; *UASwizinx3* (B) and *UASwizinx3*; *AS>*GAL4 larvae (B'). The first cross yields 50% larvae carrying both the transgene and the driver but none of them exhibit dorsal closure defects (0/115). The second cross yields 100% larvae carrying both the transgene and the driver and none of them show developmental defects. **C–E'**: Brightfield micrographs of dorso-lateral cuticle preparation of heterozygous *Df(3R)BSC789* larvae expressing *UAS-inx3::GFP* under the control of *actGAL4* (GFP positive, A), homozogous *Df(3R)BSC789* larvae not expressing *inx3::GFP* (GFP negative, B), homozogous *Df(3R)BSC789* larvae expressing *inx3::GFP* under the control of *actGAL4* (GFP positive, C and C'). Larvae in C and C' are smaller and sometimes have defects in the head or in the rear but do not exhibit any dorsal closure defects. **F**: Quantification of the rescue of the dorsal closure defects [1] by *inx3::GFP* driven *actGAL4* [2] and *AS>GAL4* [3]. The rescue cross (*w; +; Df(3R)BSC789, UAS-inx3::GFP/TM3, Sb x w; actGal4/CyO; Df(3R)BSC789/TM3, Sb)* was set. GFP positive and negative embryos were sorted before letting the larvae develop to first instar. The red color number indicates the number of larvae in [2] in which dorsal closure defects due to the deficiency are rescued by *inx3::GFP* expression under the control of *act*GAL4. Note that they represent about ½ of the GFP positive population of embryos as expected (see Materials and Methods for predicted outcome). Note that it is not the case in cross [3]. The blue color number indicated the numbers of larvae in which dorsal closure defects due to the deficiency are not rescued by *inx3::GFP* expression under the control of an amnioserosa specific driver (*AS>GAL4*). **G,G'**: Homozogous *Df(3R)BSC789* larvae expressing *inx3::GFP* under the control of *AS>GAL4* (GFP positive). They exhibit dorsal closure defects (kink (G) and holes (G')) similar to the homozogous *Df(3R)BSC789* not expressing *inx3::GFP*.

Thus, single mutant analysis for *apc*, *spg*, *wsp* and *CG1647* loci indicates that none of these genes is involved in dorsal closure. This strongly indicates the loss of Inx3 function is responsible for the dorsal closure phenotype observed in *Df(3R)BSC789* homozygous larvae. However, we cannot exclude the possibility that the combined loss of more than one of these genes leads to dorsal closure defects. Given the complexity of creating double or triple mutant combinations by recombination, we did not test this directly. Instead, to confirm that the sole loss of Inx3 is responsible for the dorsal closure defects observed in *Df(3R)BSC789* homozygous larvae, we first attempted to deplete Inx3 by RNAi by driving the expression of *UASwizinx3* transgene [35] using the UAS-GAL4 system [44]. However, in our hands, this failed to trigger a reduction in Inx3 protein levels (Figure 4A) and did not yield any phenotype (Figure 4B, B').

We then used the previously described transgenic line *UAS-inx3::GFP* [35] to monitor its ability to rescue the dorsal closure phenotype in homozygous *Df(3R)BSC789* larvae. The overexpression of inx3::GFP in *Df(3R)BSC789* homozygous embryos rescues the dorsal closure defects following the expected mendelian ratio (Figure 4F, see also materials and methods). Neither holes nor kinks were observed any longer in the dorsal epidermis (Figure 4C–E'), although inx3::GFP expression did not rescue lethality and head defects. This indicates that Inx3 expression is critical for proper dorsal closure.

**Figure 5:**
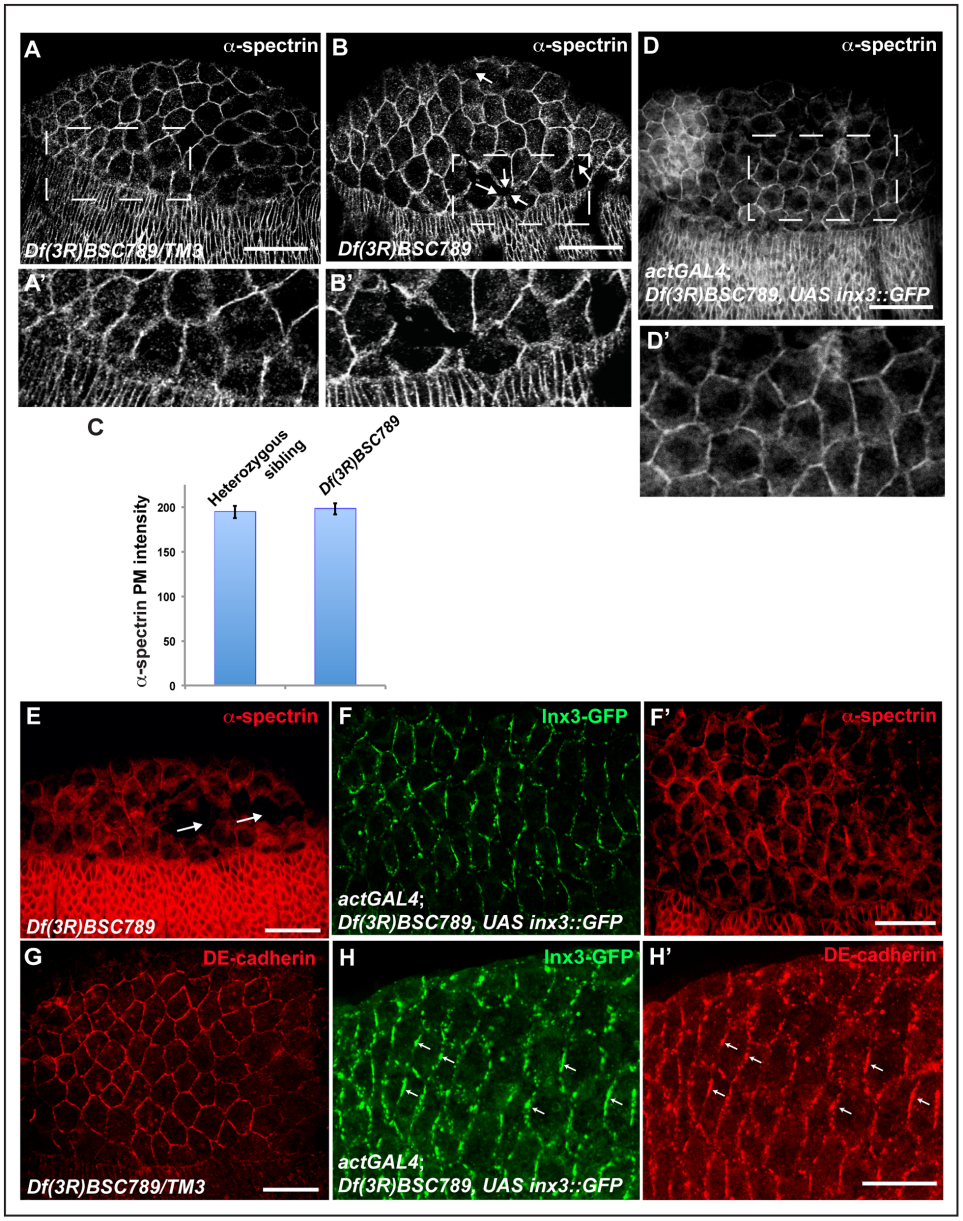
inx3::GFP overexpression rescues junctional defects in the *Df(3R)BSC789* amnioserosa. A–B: Immunolocalisation of α-spectrin in the amnioserosa and ectoderm cells outlining the cell cortex in *Df(3R)BSC789* heterozygous (A) and homozygous (B) stage 13 embryos. Note that some cell-cell junctions seem to be lost in the amnioserosa (arrows), even though the overall levels of α-spectrin are unchanged (C). A' and B' are blown up of the dashed areas in A and B, respectively. **C**: Image J quantification of the α-spectrin immunofluorescence levels as in A, B and D. **D**: Immunolocalisation of α-spectrin in *Df(3R)BSC789* mutant embryos overexpressing *inx3*::GFP under the control of *actGAL4*. None of the embryos examined (n = 44) exhibit defects in the amnioserosa. **E–F'**: Immunolocalisation of α-spectrin (E, F') and Inx3-GFP (F) in *Df(3R)BSC789* homozygous embryos (sorted GFP negative embryos) presenting loss of cell-cell contact in the amnioserosa (arrows), and the sibling *Df(3R)BSC789; inx3::GFP* (sorted GFP positive embryos). **G–H'**: Immunolocalisation of DE-cadherin (G, H') and Inx3-GFP (H) in *Df(3R)BSC789/TM3* larvae and in *Df(3R)BSC789; inx3::GFP* (sorted GFP positive embryos). Note that DE-cadherin completely co-localises with Inx3-GFP in plaques (small arrows) and looks different from the WT pattern (G). Scale bars: 25 µm.

In order to understand whether the dorsal closure defects observed in *Df(3R)BSC789* were caused by the compromised integrity of the ectoderm, amnioserosa, or both, we visualised the cell cortex with α-spectrin. In *Df(3R)BSC789* homozygous embryos, some cell-cell contacts were clearly lost in the amnioserosa, as shown by the presence of holes and gaps between cells (arrows in Figure 5B and 5E) but without changes in the overall levels of α-spectrin (Figure 5C). Conversely, no such gaps were observed in the ectoderm and the leading edge. Critically, cell-cell contact defects in the aminoserosa were also rescued by expression of inx3::GFP (Figure 5D, D', F') that localised in plaques at the plasma membrane in a manner indistinguishable from endogenous inx3 (compare Figure 5F to Figure 1C).

These results suggest that the dorsal closure defects could be explained by the loss of amnioserosa integrity, in line with its role in the morphogenetic event (see introduction). However, the expression of inx3::GFP exclusively in the amnioserosa of *Df(3R)BSC789* homozygous embryos is not sufficient to rescue dorsal closure defects (Figure 4G, G') and these embryos present the same defects as the deficiency only (Figure 3C, C' and Figure 4D). This suggests that even though there is a visible integrity phenotype in the amnioserosa but not in the ectoderm, this tissue is also affected, and Inx3 is indispensable for both these tissues for successful dorsal closure.

### The interrelation between innexins and DE-cadherin

**Figure 6:**
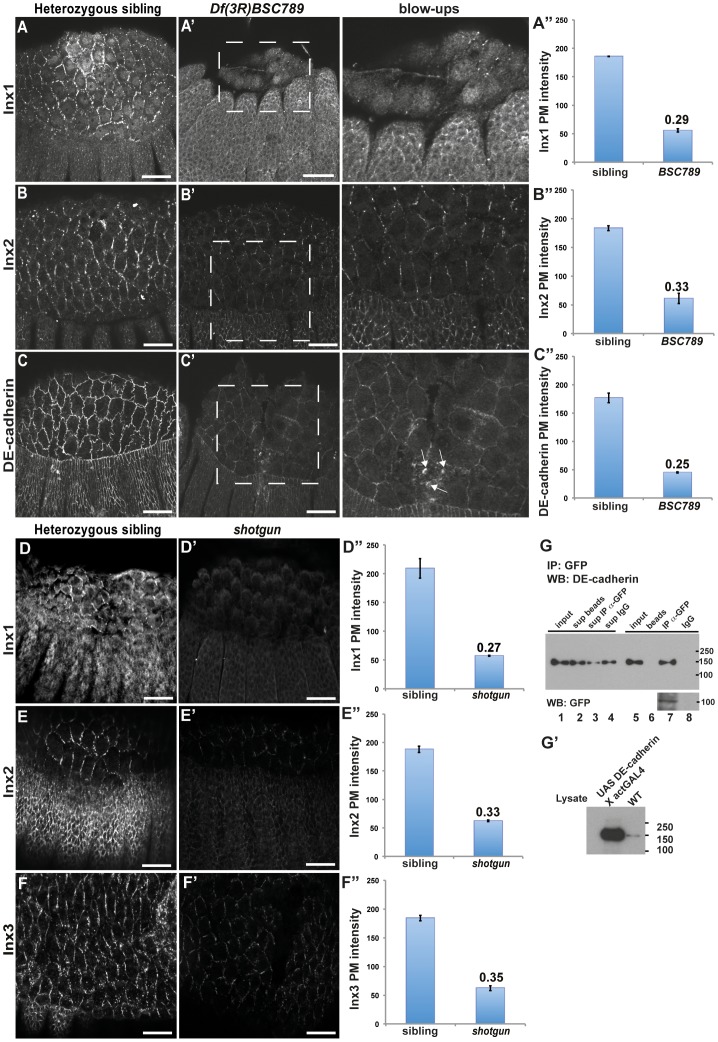
Localization defects of junctional proteins in the absence of Inx3 and DE-cadherin. A–C”: Immunolocalisation of Inx1 (A, A' and blow-up), Inx2 (B, B' and blow-up) and DE-cadherin (C, C' and blow-up) in *Df(3R)BSC789* siblings (A, B and C) and homozygous mutant embryos (A', B' and C' and inserts). Note the apical accumulation of DE-cadherin (small white arrows). Quantification of plasma membrane intensities are displayed as absolute values (A”, B” and C”). The dashed areas in A', B' and C' are blown up and the brightness has been artificially increased to reveal details. **D–F”**: Immunolocalisation of Inx1 (D, D'), Inx2 (E, E') and Inx3 (F, F') in *shotgun* heterozygous siblings (C, D and E) and homozygous mutant embryos (C', D' and E'). Quantifications of plasma membrane intensities are displayed as absolute values (D”, E” and F”). **G**: Western blot of DE-cadherin and *inx3::GFP* at different steps of immunoprecipitation of *inx3::GFP* using an anti-GFP antibody or IgG, as indicated (top panel). Sup beads, Sup IP α-GFP and Sup IgG designs the lysate supernatant after incubation with beads alone, beads coupled to the anti-GFP antibody and beads coupled to IgG, respectively. Note the presence of DE-cadherin and *inx3::GFP* bands in lane 7 and its absence in lane 8. **G'**: Western blot of DE-cadherin in lysate of OreR and *Drosophila* overexpressing DE-cadherin. Scale bars: 25 µm.

Next, we dissected the nature of the cellular defects sustaining the dorsal closure impairment in *Df(3R)BSC789* embryos (that we refer to *Df-Inx3* in the text). Given the known genetic interactions between Inx3 and other junction proteins [35], [45], the dorsal closure defects described above seemed unlikely to be due to the sole absence of zygotic Inx3 protein. We therefore examined whether loss of Inx3 might impinge on the localisation of Inx1, Inx2 and DE-cadherin in embryos at stages of dorsal closure by assessing the localization of these three proteins in *Df-Inx3* homozygous embryos. Although their localization is seemingly unaffected, we found that their levels at the plasma membrane are significantly reduced (∼3 fold) both in the ectoderm and the amnioserosa, when compared to siblings (Figure 6A–C”, Table 1). This suggests that loss of Inx3 affects the expression of Inx1, 2 and DE-cadherin both in the amnioserosa and the ectoderm. Collectively, the decreased expression of these markers would contribute to the impairment of cell-cell junctions in the amnioserosa (that loses its integrity) and to their weakening in the ectoderm respectively, hence leading to dorsal closure defects.

**Table 1:** Immunofluorescence localisation of Innexins and DE-cadherin in the amnioserosa and ectoderm in Drosophila embryos of different genetic backgrounds.

Proteins	Inx1	Inx2	Inx3	DE-cadherin
Genetic background^1^				
***Ore*** **R and siblings**	-Plaques at PM -Internal haze^2^	Plaques at PM	Plaques at PM	PM
***ogre^ko^***	-No PM staining -Internal haze^2^	as in wildtype	ND	as in wildtype
***kropf^g43^***	as in wildtype	No staining except for a a faint PM loc (probably due to the maternal pool)	as in wildtype	as in wildtype
***Df(3R)BSC789***	-Level ∼3 fold reduced at PM^3^ -Internal haze^2^	Level ∼3 fold reduced at PM^3^	No staining	- Level ∼4 fold reduced at PM^3^ +apical internal pool (possibly endo/lysosomes)^3^
***shotgun^2^***	-Level ∼4 fold reduced at PM^3^ -Internal haze^2^	Level ∼3 fold reduced at PM^3^	Level ∼3 fold reduced at PM^3^	No staining
***zipper^2^***	-Level ∼3 fold reduced at PM^3^ -Internal haze^2^	ND	-Level ∼3 fold reduced at PM^3^ - No internal pool^3^	- Level ∼2 fold reduced at PM^3^ + a strong apical internal pool (possibly endo/lysosomes)^3^

1: homozygous stage 13–14 embryos.

2: Internal haze is antibody background.

3: Same in ectoderm.

The reduced plasma membrane levels of Inx1, 2 and DE-cadherin suggest that these proteins might be degraded, prior or following their plasma membrane delivery. In this regard, we noticed a faint internal DE-cadherin domain (white arrows in Figure 6C”) that has been proposed to correspond to an endocytosed pool [46] en route for degradation in the lysosomes. This degradation could be due to the fact that in the absence of Inx3, Inx2, 1 and DE-cadherin cannot form stable complexes. In support of this hypothesis, Inx2 and 3 co-localise at the plasma membrane in the epidermis at early embryogenesis, and loss of either leads to similar phenotypes, suggesting that they genetically interact. Inx2 and 3 also interact biochemically and are proposed to form hetero-oligomers/hemichannels [35], [47]. During dorsal closure stages, however, although loss of Inx3 expression results in the loss of Inx2 in the amnioserosa and the ectoderm (Figure 6B') and yields defects, the converse is not observed. Loss of zygotic Inx2 in *kropf* mutants does not affect Inx3 (Figure S2A), and does not lead to dorsal closure defects. These findings suggest that Inx2 and 3 might form a complex in embryos at dorsal closure stages but that Inx2 is not a critical component.

So far, there is no precedent for Inx3 and Inx1 to interact, either genetically or biochemically. However, the impact of Inx3 loss of expression on Inx1 stability described above is suggestive of a genetic interaction between these two genes. Similarly for loss of Inx2 expression, loss of Inx1 expression (in *ogre* ^ko^) is not enough to affect Inx3 deposition (not shown). One possibility is that Inx1 and 2 have redundant functions. To test this, we assessed Inx3 levels and pattern in *Df(X)BSC867* (uncovering both genes) that exhibit very strong dorsal closure defects (Figure 2H). We found that Inx3 is strongly decreased in both the amnioserosa and the ectoderm (Figure 2I, I'), suggesting that the collective loss of Inx1 and 2 could, in fact, impede upon the proposed Inx3/2/1 complex formation and stability. However, this interpretation should be taken with caution as the deficiency uncovers many other genes (see above) that might have a role in the destabilization of this complex. Taken together, these data indicate that Inx3, Inx2 and perhaps Inx1 bind each other at the plasma membrane of amnioserosa and ectodermal cells during dorsal closure stages to form heterologous channels.

Inx3 and DE-cadherin mutually influence each other's expression. As in the epidermis of early embryos [35], Inx3 influences DE-cadherin dynamics and stability at the plasma membrane (Figure 6C–C”), and, conversely, loss of DE-cadherin zygotic expression (in *shotgun*) affects the plasma membrane levels of Inx3 (Figure 6F, F', Table 1). This observation suggests that they might physically interact. To test this hypothesis biochemically, we performed immunoprecipitation of Inx3-GFP with an anti-GFP antibody followed by western blotting with an anti-DE cadherin that recognises a specific band at 150kd (Figure 6G'). We found that DE-cadherin was specifically pulled down with Inx3-GFP (Figure 6G, compare lanes 7 and 8), showing that they form a complex. Furthermore, Inx3-GFP expression in the *Df-Inx3* homozygous embryos results in the increase of DE-Cadherin levels (when compared to the not rescued deficiency (Figure 6C') but interestingly, the DE-Cadherin distribution differs from the one observed in the wildtype (Figure 5G), as it appears exclusively recruited to the inx3::GFP expression domain at the plasma membrane (Figure 5G-H', arrows). This further reinforces the notion that Inx3 and DE-cadherin form a complex, possibly upon reaching the plasma membrane where they contribute to their reciprocal stabilisation.

This finding is in line to the reported interaction between Inx2 and DE-cadherin in epidermis of early embryos [45]. To assess whether DE-cadherin and Inx2 also interact during dorsal closure stages, we monitored Inx2 protein levels in *shotgun* homozygous embryos and found that its protein levels at the plasma membrane are very reduced (Figure 6D, D'), indicating that DE-cadherin is required for Inx2 maintenance. However, DE-cadherin expression was unaffected in *kropf* mutant embryos (Figure S2B, Table 1). Similarly, DE-cadherin was unaffected in *ogre* ^ko^ homozygous embryos, whereas Inx1 was severely affected in *shotgun* mutant embryos. This suggests, as above, that Inx1 and 2 might have redundant functions to modulate DE-cadherin stability. To test this, we monitored DE-cadherin distribution in *Df(X)BSC867* and find it strongly affected both in the ectoderm and the amnioserosa (Figure 2J, J'), suggesting that the combined loss of inx1 and 2 impedes on adherens junctions. As above, however, caution needs to be exerted as the deficiency uncovers many genes that could lead to the destabilization of DE-cadherin. Taken together, these data indicate that Inx3/2/1 complex interacts with DE-cadherin, in line with the fact that gap junctions are embedded into adherens junctions. These interactions contribute to junctional stability that is necessary for proper dorsal closure. In this complex, Inx3 has a prominent role and its loss of function cannot be compensated by the presence of either Inx1 or Inx2. This shows that Inx3 is essential, at least at dorsal closure stages, and we propose that Inx3 is genetically upstream of Inx1 and 2.

DE-cadherin has also a critical role and, as it is considered the master regulator of all junctions, impairment of Inx1 and 2 stabilisation in *Df-Inx3* homozygous mutant embryos could also be a consequence of DE-cadherin mislocalisation and degradation, not only of the loss of Inx3 expression.

### Inx3 stability depends on tissue tension

That loss of DE-cadherin [48], [46], [49], [50], [51], [52], [53], [19] and Inx3 expression (this manuscript) leads to dorsal closure defect indicates that cell-cell junctions are critical in dorsal closure. Junctions have a role in maintaining tissue integrity, and, due to their binding to the cell cytoskeleton (especially actin), they also ensure the appropriate tissue tension required for dorsal closure, in allowing contraction of the amnioserosa and the migration of the ectoderm. In this regard, when junctional strength increases, as a consequence of Crumbs overexpression [18] or in the case of upregulation of septate junction genes by ectopic expression of *grainyhead* in the amnioserosa [6], dorsal closure is impaired.

Conversely, tissue tension itself feeds back to the trafficking and stability of junctional molecules, especially E-cadherin. In this regard, E-cadherin (but not desmosomal cadherin) is lost from adherens junctions in keratinocytes depleted of two key actin remodeling small GTPases, RhoA and Rac, [54], [55]. In *Drosophila*, Rho1 has also been implicated in the regulation of DE-cadherin localization. Loss of Rho1 function leads to DE-cadherin mislocalization with an ectopic cytoplasmic accumulation, thought to correspond to an endocytosed pool [50], [46]. Conversely, dominant-negative Rho1 reduces DE-cadherin in adherens junction, resulting in dorsal closure with specific leading edge disorganisation [48].

**Figure 7:**
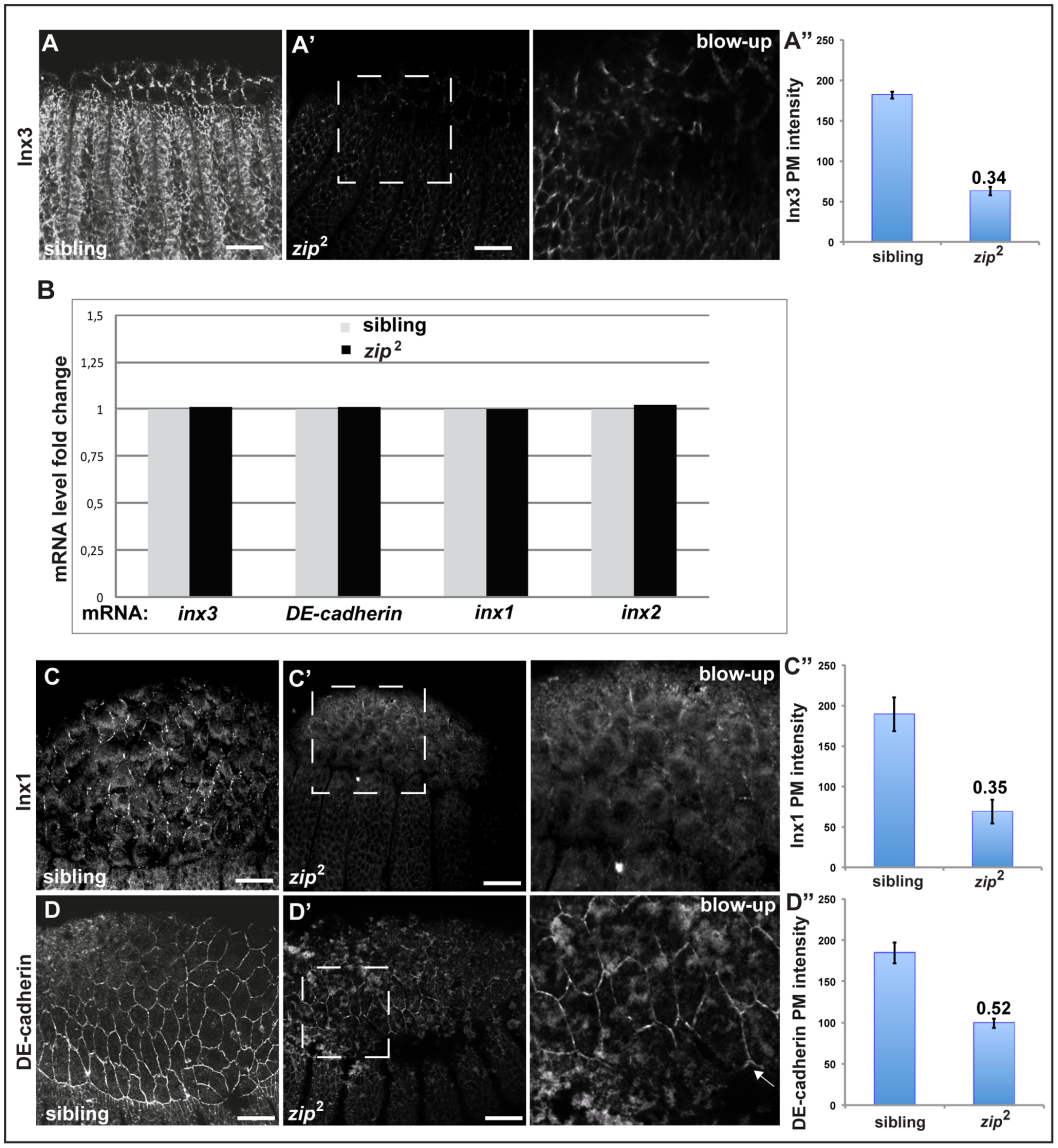
Expression and localization of junctional proteins in *zipper.* A–A”: Immunolocalisation of *nx3* in heterozygous siblings (A) and homozygous *zip* ^2^ mutant embryos (A' and insert). Quantifications of plasma membrane intensities are displayed as absolute values (A”). **B**: Real time PCR of *inx3*, *shg*, *inx1* and *inx2* mRNA in heterozygous siblings and homozygous *zip*2 mutants. **C–D”**: Immunolocalisation of Inx1 (C, C' and insert) and DE-cadherin (D, D' and insert) in heterozygous siblings (C, D) and homozygous *zip2* mutants (C', D' and inserts). Inserts are blown ups of the dashed areas in A', B' and C' and the brightness has been artificially increased to reveal details. Quantifications of plasma membrane intensities are displayed as absolute values (C” and D”). Scale bars: 25 µm.

Since Inx3 localisation to the plasma membrane is critical for the dynamics of the other innexins and DE-cadherin, this prompted us to study Inx3 expression and dynamics during the stages of dorsal closure with respect to tissue tension. For this purpose, we used the mutant allele for *zipper*, *zip^2^*. *zipper* encodes the non-muscle myosin II heavy chain, a critical subunit of MyoII that, once activated by ROCK phosphorylation, is responsible for apical contraction of amnioserosa cells and the migration of the leading edge ectodermal cells [56], [57], [58]. Strikingly, we found that in *zip^2^* homozygous mutant embryos, Inx3 levels at the plasma membrane were severely reduced at the plasma membrane of the amnioserosa and ectodermal cells (Figure 7A).

This result can be interpreted in two ways: either expression of Inx3 is reduced at the transcriptional level in line with gene expression regulation by tissue tension, or Inx3 is normally expressed but is degraded before or after it reaches the plasma membrane. To test the first possibility, we monitored *inx3* mRNA expression by real-time PCR in *zip^2^* mutant embryos, but found that its mRNA levels are identical to heterozygous siblings (Figure 7B). This shows that *inx3* gene expression is not sensitive to tissue tension and that the decrease in Inx3 protein levels are likely to be due to its post-translational degradation. In agreement with the *Df-Inx3* phenotype, Inx1 (Figure 7C–C”) and 2 (data not shown) plasma membrane levels are also decreased in *zip* ^2^, presumably as a result of the loss of Inx3.

We also monitored *shg* mRNA expression and protein localization in *zip* ^2^. *shg* mRNA levels are similar in homozygous *zip* ^2^ embryos when compared to heterozygous (Figure 7B). Furthermore, although in *zip* ^2^, DE-cadherin exhibits an ectopic cytoplasmic accumulation, possibly corresponding to endocytosis, as previously reported for the *rho1* mutant [50], [59], the amount of DE-cadherin at the plasma membrane is only less than 2 fold reduced when compared to the heterozygous siblings (Figure 7D–D”). This result shows that Inx3 stability is directly triggered by tissue tension independently (at least partly) of DE-cadherin. Furthermore, this reinforces the finding that Inx1 and 2 maintenance at the plasma membrane is dependent on Inx3 as well as of DE-cadherin, putting Inx3 upstream of Inx1 and 2.

In conclusion, we show for the first time that three members of gap junction proteins Innexin1, 2 and 3 are expressed in the amnioserosa and ectoderm during dorsal closure stages. We also provide genetic evidence for the existence of a hierarchy of molecular interactions among these three markers that is critical for proper dorsal closure. Contrarily to zygotic loss of *inx1* and *2* expression that has no effect on dorsal closure on their own, loss of *inx3* expression leads to dorsal closure defects deriving from loss/weakening of cell-cell junction by severely affecting the plasma membrane levels of the Inx1, 2 and DE-cadherin. Last, our data support a model in which Inx3 acts in parallel to DE-cadherin and upstream of Inx1 and 2 for proper maintenace of the amnioserosa and ectoderm integrity in response to tissue tension during dorsal closure.

## Materials and Methods

### Fly culture and stocks

Flies were raised at 25°C under standard conditions unless otherwise stated. *OregonR* line was used as the wildtype reference strain and is referred to as WT.

*kropf^g43^*, *shg^2^* [8], *zip^2^* [56], *Df(1)BSC867*, *Df(3R)BSC789*, *Df(3R)BSC806, spg^5^*, *CG1647^EY05613^* and *apc^MI01007^* were obtained from Bloomington Stock Center. The *wsp^1^* line was kind gift from Patrik Verstreken (Leuven, Belgium).

### Homologous recombination

Ends-out homologous recombination at the ogre locus (Chromosome X:6867648-6885351) was performed as described [38]. Two arms (∼4kb each) obtained from genomic PCR were subcloned into the pW25 vector to create the construct *ogre* ^HR^-pW25 used for generating transformants (Bestgene, CA). Transgenic flies bearing this construct on chromosome 2 were crossed to yw; 70FLP, 70I-SceI, Sco/CyO flies, and the progeny (F_1_) were heat-shocked at 37°C for 1h at day 3, after egglaying. The *ogre^HR^-pW25*/70FLP, 70I-SceI, Sco virgins of the F_1_ progeny were crossed to w1118 males. Approximately 0.03% of the total F_2_ progeny displayed red eyes (6 of ∼18,800 flies). Two independent lines showed translocation of the w^+^ marker from chromosome 2 to X. Targeted homologous recombination was verified by genomic PCR and sequencing using *ogre* forward primers (5′-GCT CCT CCG GGG ATT GGG CT-3′) and *ogre* reverse primers (5′-GCCACACCGGGATGAGCCAC-3′).

### UAS-inx3::GFP rescue of Df(3R)BSC789

*UAS-inx3::GFP* transgene [35] was recombined with *Df(3R)BSC789.* Overexpression of *UAS-inx3::GFP* was accomplished by the Gal4-UAS system [44] making use of the ubiquitous driver *actinGAL4/CyO* (*actGAL4*) or the amnioserosa specific driver *c^381^GAL4* (referred to as *AS>GAL4* in the legend for figures) (Bloomington Stock Center). Briefly, *Df(3R)BSC789, UAS-inx3::GFP/TM3, Sb* flies were crossed to *actGal4/CyO; Df(3R)BSC789/TM3, Sb*. For the amnioserosa specific rescue experiment, *Df(3R)BSC789*, *UAS-inx3::GFP*/TM3, Sb flies were crossed to *Df(3R)BSC789*/TM3, Sb; *c^381^GAL4*.

To quantify the rescue of the dorsal closure of *Df(3R)BSC789* by overexpression of inx3::GFP, GFP positive embryos (1/4 of the laid embryos) were sorted under a binocular and either grown as first instar for cuticle preparations to score their development, or fixed and processed for (immuno)fluorescence for assessing the tissue integrity. In Table 2, the predicted outcome of the rescue cross using the *act*GAL4 is listed (in bold is the genotype corresponding to the rescued larvae marked in red in Figure 4F).

**Table 2:** Predicted outcome of the rescue cross using the *act*GAL4.

Genotype	GFP	Expected ratios
*w; actGal4/+; Df(3R)BSC789, UAS-Inx3::GFP/TM3, Sb*	positive	12.5% (50% of GFP pos)
***w; actGal4/+; Df(3R)BSC789, UAS-Inx3::GFP/Df(3R)BSC789***	**positive**	12.5% (**50% of GFP pos**)
*w; actGal4/+; Df(3R)BSC789/TM3, Sb*	negative	12.5%
*w; actGal4/+; TM3, Sb/TM3, Sb*	negative	12.5%
*w; +/CyO; Df(3R)BSC789, UAS-Inx3::GFP/Df(3R)BSC789*	negative	12.5% (DC phenotype: 16.6% of GFP neg)
*w; +/CyO; Df(3R)BSC789/TM3, Sb*	negative	12.5%
*w; +/CyO; Df(3R)BSC789, UAS-Inx3::GFP/TM3, Sb*	negative	12.5%
*w; +/CyO; TM3, Sb/TM3, Sb*	negative	12.5%

Expected number of progeny of the different genotypes from the following rescue cross: *w; +; Df(3R)BSC789, UAS-Inx3::GFP/TM3,Sb* x *w; actGal4/CyO; Df(3R)BSC789/TM3,Sb.*

### RNAi depletion of Inx3

To knockdown Inx3 by RNAi, the *UASwizinx3* line [35] was crossed to the ubiquitous driver *actGAL4*/CyO and the amnioserosa-specific driver *c^381^* on chromosome 4 (Bloomington Stock Center), at 29°C as previously described in [35].

### Cuticle preparations

Cuticles were prepared as described by [60] with minor changes. Eggs were collected for 6h at 25°C on yeasted grape juice plates and aged an additional 24 h. They were dechorionated in 50% bleach for 5 min, and rinsed with water, NaCl/Triton and three times in 0.1% Triton-X 100. Embryos were then transferred into an eppendorf containing 0.5 ml methanol and 0.5 ml heptane and shaken vigorously for a period of time of 1 min to allow the removal of the vitelline membrane. Embryos that sunk to the bottom of the tube were mounted with mixture of lactic acid and Hoyer's mountant (1:1) on glass slides and topped with a coverslip. Mounted slides were placed at 60°C overnight to allow clearing.

### Immunofluorescence microscopy

Stage 12–15 embryos from different lines were collected on yeasted grape juice plates for a period of 1–2h at 25°C and let to develop at 25°C for 9 additional hours. Embryos were dechorionated in bleach for 3 min, rinsed thoroughly with NaCl/triton, fixed with 37% formalin (formaldehyde in 10% methanol) for 5 min and stored at −20°C in 100% methanol. For DE-cadherin labeling with the DCAD2 antibody, embryos were fixed in 4% formalin for 20 min and store at −20°C in 100% ethanol, as described in [61].

Immunofluorescence was performed as previously described [62]. The following primary antibodies were used: mouse anti α-spectrin 3A9 (1:20, Developmental Studies Hybridoma Bank), rabbit anti-Inx1 (1:50) [34], rabbit anti-Inx2 (1:50) [34], rabbit anti-Inx3 (1:40) [35], rat anti-DE-cadherin DCAD2 (1:100; Developmental Studies Hybridoma Bank). FITC- and TRITC-conjugated secondary antibodies (Invitrogen) were used to recognize the primary antibodies. Samples were mounted in ProLong Gold antifade reagent (Invitrogen) on glass slides. Images were acquired using 40x objective on a Leica SPE2 confocal microscope.

### Estimation of immunofluorescence intensity of junction proteins at the plasma membrane

The measurement of the fluorescence intensity of junction proteins was performed using images captured at identical confocal settings. Stacks of images were taken and the three slices showing the brightest labeling were used for the measurements. Average pixel intensities for 10 junctions per embryo (on 3 confocal slices), 3 embryos of each genotype, were calculated in Image J using the line measurement tool set to a six-pixel width and background from a laser-off image was subtracted. Sibling and mutant mean intensities were averaged and then statistically compared using an unpaired two-tailed Student's t-test.

### Co-immunoprecipitation

For co-immunoprecipitation, staged embryos overexpressing *UAS-inx3::GFP* under the control of actin GAL4) were selected under a fluorescence binocular and homogenized for 15 minutes on ice in lysis buffer (10mM HEPES at pH 7.9, 5mM MgCl_2_, 0.5% NP-40, 0.45M NaCl, 1mM DTT) containing the complete protease and phosphatase inhibitor cocktails (Roche). Protein lysate was first incubated with 4µg of rabbit anti-GFP antibody (Chemokine) for 3 h followed by 1h incubation with protein A sepharose (Pierce). Bound protein was released from pelleted protein A by direct incubation with 2% SDS loading buffer and 40μl of supernatants were analyzed according to standard western blotting techniques. DCAD2 antibody (Developmental Studies Hybridoma Bank) was used at a concentration of 1:50 for DE-cadherin detection.

### Real-time RT-PCR

RNA was extracted with TRIZOL reagent (Invitrogen) according to the manufacturer's instructions. Then, 5 µg of total RNA was used to perform reverse transcription (RT) for first-strand cDNA synthesis. RNA was denatured for 5 min at 70°C and snap cooled on ice in the presence of 0.5 µg Oligo(dT). Next, it was GoScript 5xReaction, 1.5 mM MgCl_2_, and 10 mmol dNTP mix, 20 units of RNasin Ribonuclease Inhibitor and 100 units of GoScript Reverse Transcriptase were added to a final volume of 20 µl and the reaction was incubated for 50 min at 42°C. Each reaction was stopped by incubation at 70°C for 15 min. All of the reagents used for RT were from Promega.

Real-time RT-PCR was performed using the IQ SYBR^®^ Green Supermix (Biorad). We quantified the transcripts of the *actin5C* gene as a control. Primers used are listed in Table 3.

**Table 3:** Primers used for real time RT-PCR.

	*Forward primers*	*Reverse primers*
*inx1*	5′-GGCCAATGATTTTGGCGA-3′	5′-TCGTGATATTCAGACCCATCAC-3′
*shg*	5′-GACGTTTGCACCTTCAACGT-3′	5′-CCGCAGAATCTCGTATTCGA-3′
*inx3*	5′-ACAAGGCGGTCATCGACA-3′	5′-GTGTAGGTGATCCAGCAGAAGG- 3′
*inx2*	5′-AGCATAGCGCCCACAAGC-3′	5′-TATCCTTTATCCTCCCTCCGC-3′
*actin5C*	5′-CCATTGAGCACGGTATCGT-3′	R 5′-GTCATCTTCTCACGGTTGGC-3′

PCR was carried out after incubation at 50°C for 2 min and pre-denaturing at 95°C for 3 min, followed by 40 cycles at 95°C for 30 sec and 62°C for 1 min. The relative quantification was given by the CT values, determined by triplicate reactions for all of the samples for *inx1, inx2, inx3*, *shotgun* and *actin5C*. The triplicate CT values of *inx1, 2* and *3* were averaged, and the CT value of *actin5C* was subtracted to obtain ΔCT. The relative expression levels of *inx1, 2, 3* and *shotgun* mRNAs were determined as 2^(−ΔCT)^.

## Supporting Information

Figure S1 **Innexin1, 2 and 3 localize to the plasma membrane of amnioserosa cells and the ectoderm during dorsal closure stages.** A-C: Immunolocalisation of endogenous Inx1 (A), Inx2 (B) and Inx3 (C) in OreR *Drosophila* embryos (stage 11–15) using specific antibodies as in Figure 1. Scale bars: 25 µm.
(PDF)

Figure S2 **Localisation of junctional proteins in different genetic backgrounds.** A–B: Immunolocalisation of endogenous Inx3 (A) and DE-cadherin (B) in *kropf* ^g43^ mutant embryos. Scale bars: 25 µm.
(PDF)
