# Supplementary figures and images for: Innexin 3, a New Gene Required for Dorsal Closure in Drosophila Embryo
Source: PLoS One. 2013 Jul 24;8(7):e69212. doi: 10.1371/journal.pone.0069212 (PMC3722180; doi:10.1371/journal.pone.0069212)

Supplementary Figure S1: Giuliani et al, 2013 PONE-D-12-36772R1

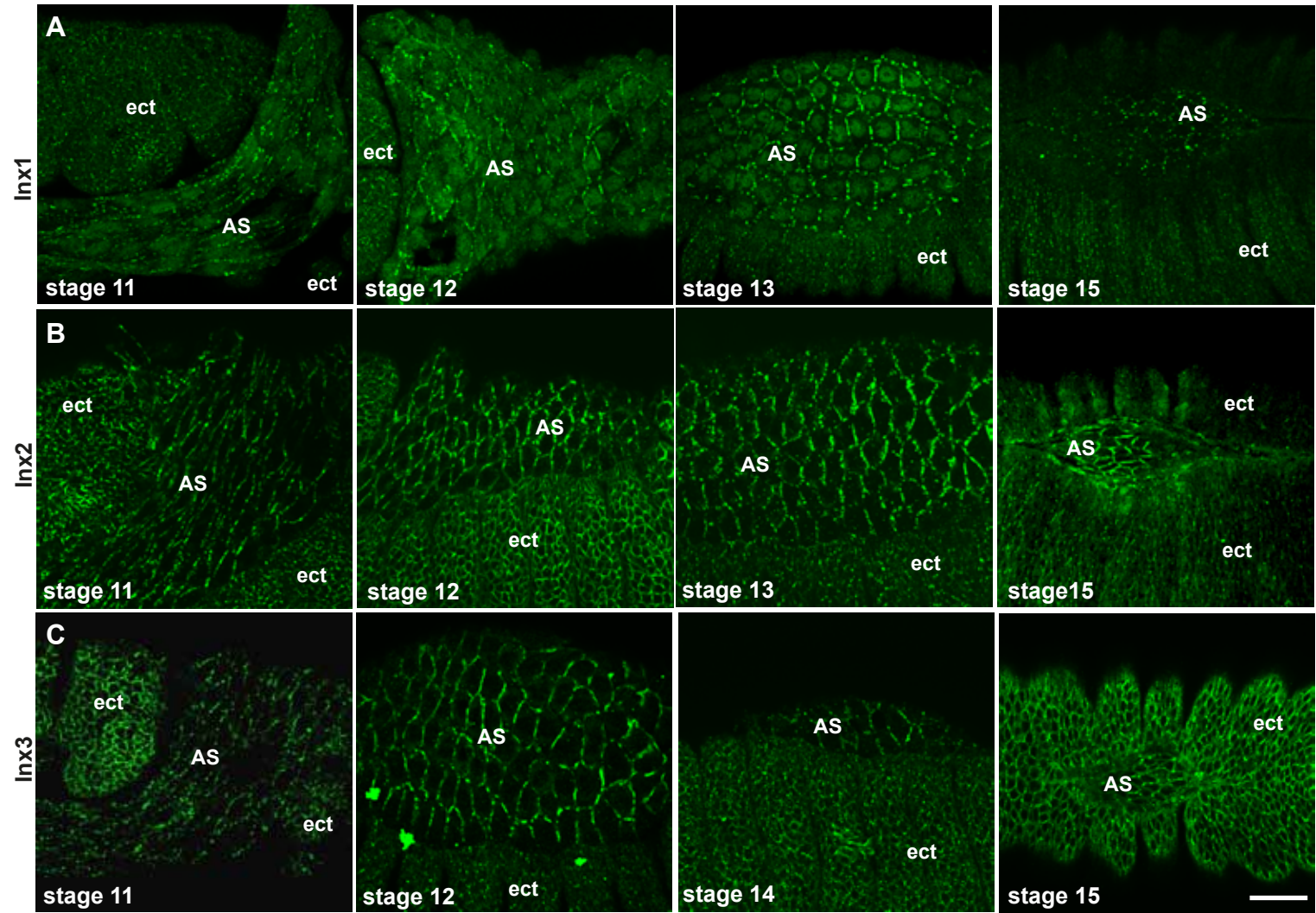

Supplement: Figure S1 — Innexin1, 2 and 3 localize to the plasma membrane of amnioserosa cells and the ectoderm during dorsal closure stages. A-C: Immunolocalisation of endogenous Inx1 (A), Inx2 (B) and Inx3 (C) in OreR Drosophila embryos (stage 11–15) using specific antibodies as in Figure 1. Scale bars: 25 µm. (PDF) [file pone.0069212.s001.pdf]

Supplementary Figure S2: Giuliani et al, 2013 PONE-D-12-36772R1

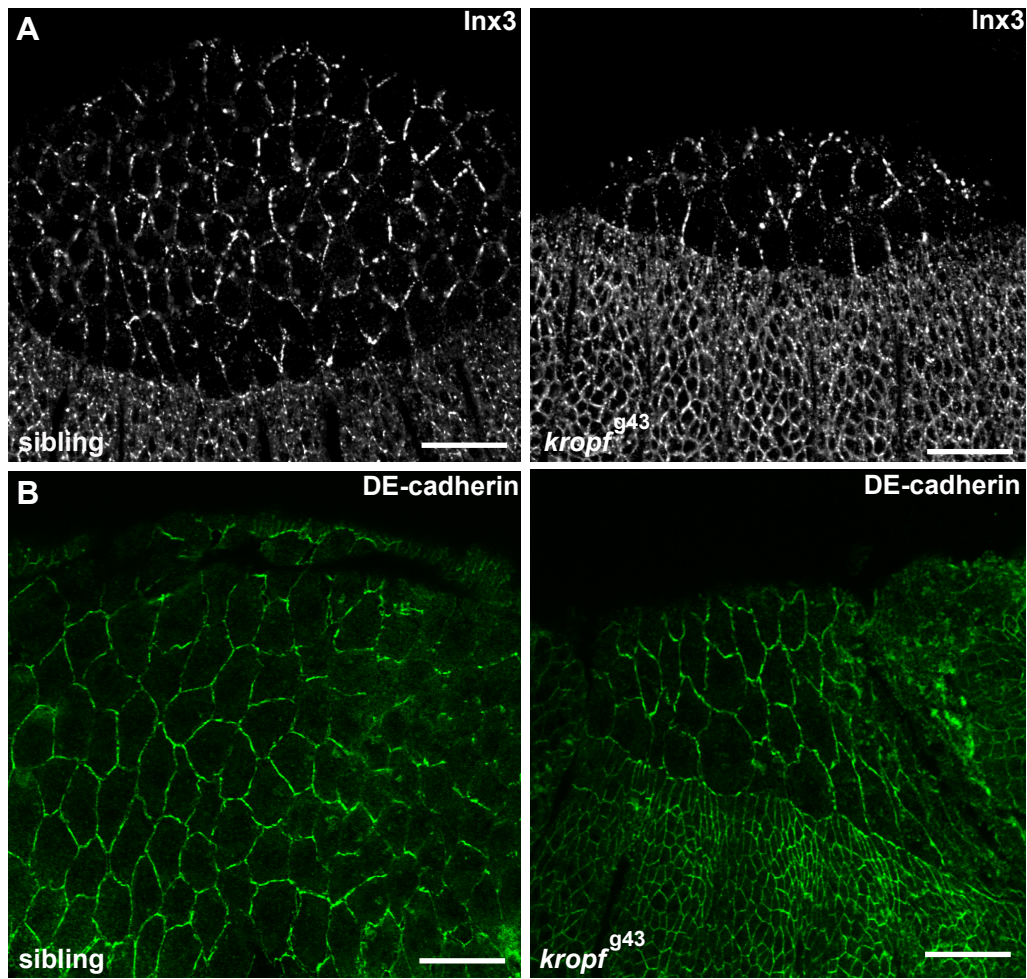

Supplement: Figure S2 — Localisation of junctional proteins in different genetic backgrounds. A–B: Immunolocalisation of endogenous Inx3 (A) and DE-cadherin (B) in kropf g43 mutant embryos. Scale bars: 25 µm. (PDF) [file pone.0069212.s002.pdf]
